# Supplementary material for: Content-rich biological network constructed by mining PubMed abstracts
Source: BMC Bioinformatics. 2004 Oct 8;5:147. doi: 10.1186/1471-2105-5-147 (PMC528731; doi:10.1186/1471-2105-5-147)
Supplement: Additional File 5 — The original Chilibot query results of the term "long-term potentiation (LTP)" and 22 other terms, limiting the latest references analyzed to the years 1990, 1995, 2000, and 2004. [file 1471-2105-5-147-S5.bz2 › chilibotAdditionalFile5/ltp2000/html/TRKA_ZIF268.html]

 


 **TRKA** and **ZIF268** 
  
Found 2 abstracts in PubMed,  **2 abstracts were retrieved and analyzed**.  


---

 Search Google  |
 PDF files only 
|  EDU domain only 

---

**Interactive relationship** (e.g. stimulation, inhibition, etc)

- However, the  **TrkA**  isoform containing the variable exon showed significantly higher activation by NT 3, which was detected by stimulation of  **TrkA**  autophosphorylation, induction of  **ZIF268**  transcription, and cellular differentiation.  Ref: 7972023 Proc Natl Acad Sci U S A, 1994

- :-)
